# Towards More Practical Methods for the Chemical Synthesis of Thioamides Using Sulfuration Agents: A Decade Update

**DOI:** 10.3390/molecules28083527

**Published:** 2023-04-17

**Authors:** Qiang Zhang, Laurent Soulère, Yves Queneau

**Affiliations:** 1 Hubei Key Laboratory of Purification and Application of Plant Anti-Cancer Active Ingredients, Hubei University of Education, 129 Second Gaoxin Road, Wuhan 430205, China; qiang.zhang@hue.edu.cn; 2 Univ Lyon, INSA Lyon, Université Claude Bernard Lyon 1, CNRS, UMR5246, ICBMS, Institut de Chimie et de Biochimie Moléculaires et Supramoléculaires, Bât. E. Lederer, 1 rue Victor Grignard, F-69622 Villeurbanne, France

**Keywords:** thioamide, sulfur, sulfuration agent

## Abstract

Compounds possessing a thioamide function play a crucial role in organic synthesis, serving as key building blocks. They are also important in the pharmaceutical chemistry and drug design, owing to their ability to mimic the amide function in biomolecules while retaining or developing biological activity. From the synthetic viewpoint, several methods have been developed for preparing thioamides using sulfuration agents. The purpose of this review is to give an update of the last decade of contributions focusing on the formation of thioamides employing different sulfur sources. When appropriate, the cleanness and practicality of the new methods are highlighted.

## 1. Introduction

**Figure 1:**
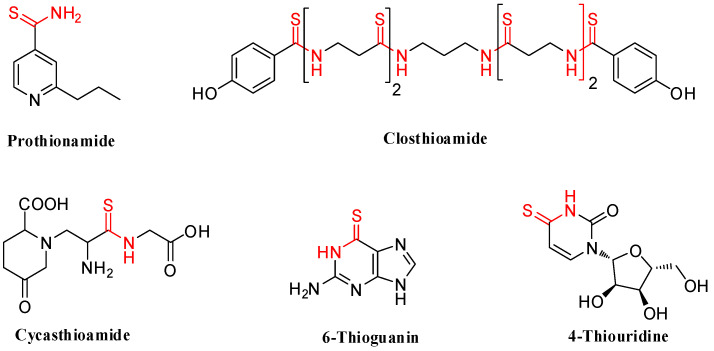
Representative pharmaceuticals or natural products containing a thioamide scaffold.

The thioamide function is one of the vital structural components present in many natural products and pharmaceutical molecules [1,2,3], such as prothionamide [4], closthioamide [5], cycasthioamide [6], 6-thioguanine [7], and 4-thiouridine [8] (Figure 1). Naturally occurring thioamide-containing peptides being important biomolecules, the chemical pathways enabling the biosynthesis of thiopeptides in different organisms have been investigated [9]. The thioamide functional group, as a bioisostere of the amide bond, has been associated with enhanced chemical stabilities and improved biological activities of pharmaceuticals compared with the corresponding molecule-bearing amide functions [10]. Rationales for these effects have been proposed, discussing notably the hydrogen bonding strength and the structural impact of thioamide backbone modifications [11,12].

The usefulness of thioamide-containing molecules relies also on their ability to serve as reactive intermediates towards various heterocyclic compounds by reaction with dielectrophilic agents [13]. Owing to different reactive centers in the thioamide function, their heterocyclization reaction may lead to the formation of thiazoles [14], thiazolines [15], thiazines [16], and benzothiazoles [17,18]. 

**Figure 2:**
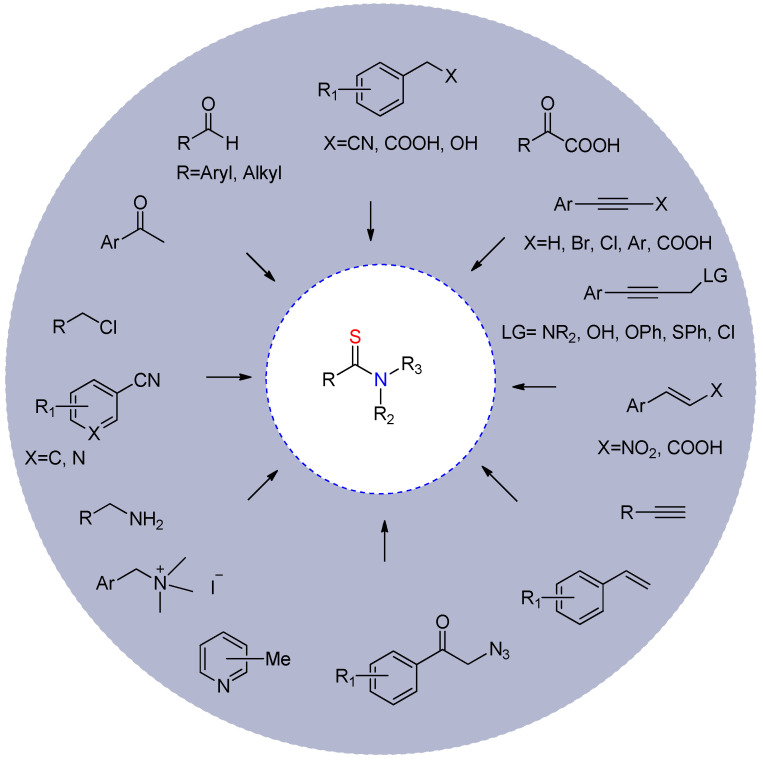
Applicable substrates to synthesize thioamides.

Thioamide compounds have therefore attracted considerable attention from organic chemists, and recent years have been rich in reported novel protocols looking for better practicality, efficiency, or environmentally friendliness. A wide range of starting materials have been employed to construct thioamides, including aldehydes, alkynes, alkenes, amines, benzyl halides, phenyl acetonitrile, cinnamic acids, α-azido ketones, and several others, as depicted in Figure 2.

The purpose of this review is to give an overview of the synthetic routes towards thioamides published in the last decade or so, giving to organic chemists a practical tool for their design and their investigation. For Lawesson’s reagent or P_4_S_10_ as thionation methods, readers can rely on two comprehensive reviews [19,20]. This account will focus mainly on all other methods employing sulfuration agents including elemental sulfur and inorganic sulfide, which often offer several advantages over the classical Lawesson’s reagent- or P_4_S_10_-mediated reactions in terms of practicality, selectivity, toxicity, and severe reaction conditions. 

## 2. Elemental Sulfur as a Sulfuration Agent

**Figure 3:**
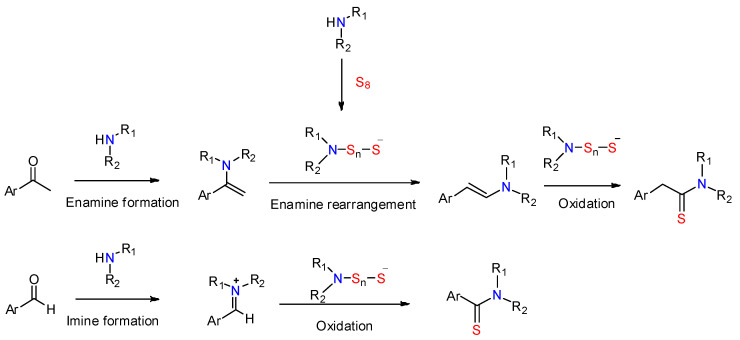
The mechanism of the Willgerodt–Kindler reaction starting from ketone or aldehyde.

Elemental sulfur (S_8_) has been extensively utilized for O-S exchange reactions or C-S bond formations, which can play the roles of reagent, oxidant, reducing agent, or catalyst depending on the specific process. The typical Willgerodt–Kindler reaction involves the oxidation/rearrangement of a ketone by using elemental sulfur and primary or secondary amines, leading to the thioamide linkage. When aldehydes are used as the carbonyl substrate instead of ketones, the reaction does not involve the rearrangement step (Figure 3). 

Though a comprehensive review on the Willgerodt–Kindler reaction was reported in 2013 [21], important updates over the last decade on this reaction and their variations are worth mentioning. Indeed, the classical reaction conditions, often performed in organic solvents and at high temperature, can however exhibit moderate yields and lead to complex mixtures. In order to be efficiently applied to the synthesis of complex compounds of pharmaceutical interest, the challenge for this reaction is therefore to find practical conditions that allow reaching higher yields, while exhibiting high functional group tolerance.

### 2.1. Characteristics and Practical Issues

Elemental sulfur is nontoxic to humans, naturally abundant, easily available with high purity, stable under ambient conditions, insoluble in water, and easy to handle. It is therefore a reagent of choice which provides a pathway to develop new protocols in the synthesis of thioamide. The reactivity of elemental sulfur relies normally on the additional presence of a chemical activating agent [22]. Reactions using elemental sulfur generally require very easy and classical work-up, involving washing the crude organic layer with water or a NaHCO_3_ solution, followed by column chromatography purification.

### 2.2. Thionylation of Aldehydes, Ketones, and Acids 

**Scheme 1:**
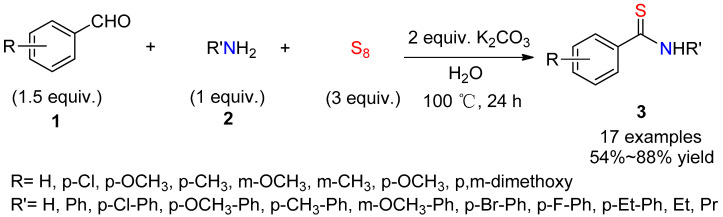
K_2_CO_3_-mediated synthesis of aromatic thioamides in water by Zhou and co-workers [23].

A three-component synthesis of aromatic thioamides **3** was established in a one-pot procedure, which involved substituted benzaldehydes **1**, primary amines **2**, and elemental sulfur (Scheme 1) [23]. The reaction is performed under catalyst-free and no-organic-solvent conditions, providing thus a range of desired aryl-substituted thioamides in a very practical way.

**Scheme 2:**
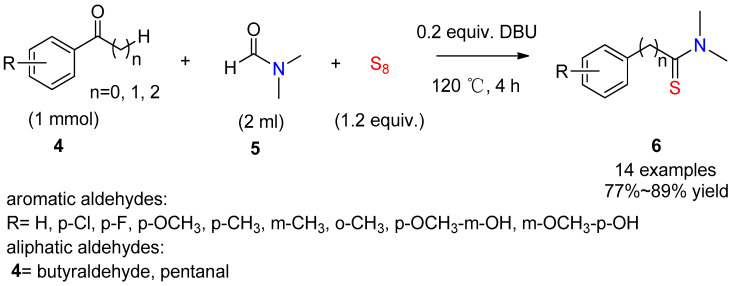
DBU-mediated synthesis of *N*,*N*-dimethyl thioamides in DMF by Liu and co-workers [24].

DMF can act as both solvent and reactant in this transformation, as exemplified for the Willgerodt–Kindler reaction involving either variously substituted benzaldehydes or acetophenones **4** (Scheme 2). In this conversion, the key intermediate dimethylamine originated from a base-mediated cleavage of DMF. Compared with the established methods, this route provides a straightforward and rapid protocol to access *N*,*N*-dimethyl thioamides **6** [24]. 

**Scheme 3:**
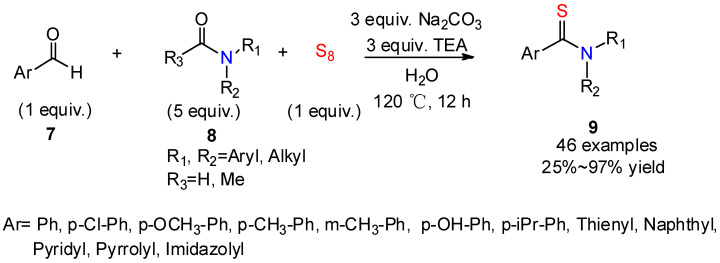
Mixed bases promoted synthesis of aryl thioamides in water by Yuan and co-workers [25].

This protocol is extendable to other amides. For example, Yuan’s group [25] recently reported a thioamidation reaction using various amides as amine sources in water (Scheme 3). Screening different organic bases, inorganic bases, or their mixtures, they found the combination of sodium carbonate and triethylamine afforded the desired aryl thioamides **9** in a high yield. Their study of the scope of formamides and aryl aldehydes demonstrated that the method was compatible with many functional groups, including halides and heteroaromatic rings. Interestingly, this protocol avoided the use of an excess of *N*-substituted formamide or acetamide.

**Scheme 4:**
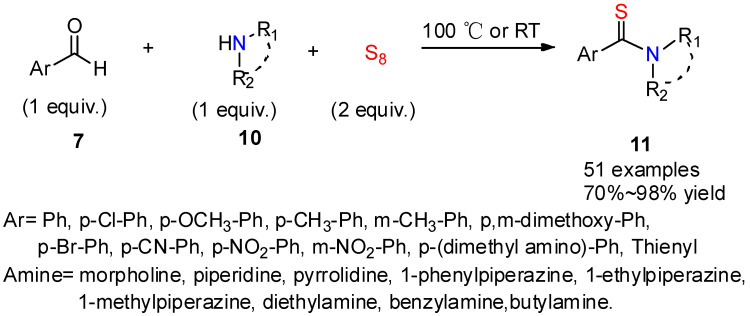
Catalyst- and solvent-free synthesis of aryl thioamides by Dalal and co-workers [26].

A catalyst-free and solvent-free Willgerodt–Kindler reaction was developed by Dalal’s team to prepare aryl thioamides **11** in good yields (Scheme 4). The reaction was performed at 100 ℃ using cyclic secondary amines including pyrrolidine, piperidine, and morpholine [26]. Some acyclic amines were also explored, such as diethylamine, which led to the corresponding aryl thioamides in good-to-excellent yields at room temperature. This method is remarkable for its clean reaction conditions which avoid the use of organic solvent and catalyst.

**Scheme 5:**
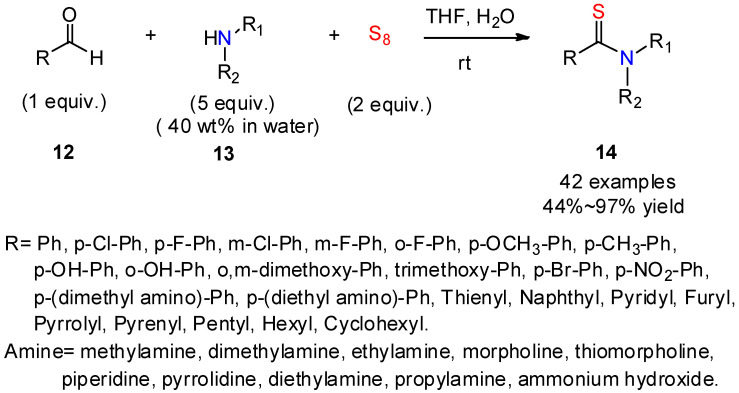
Water-mediated synthesis of aryl/alkyl thioamides by Gururaja and co-workers [27].

A catalyst-free protocol using alkyl or aryl aldehydes toward thioamides **14** was reported by Gururaja’s group (Scheme 5) [27]. The reaction can proceed in water with small amounts of THF, in the absence of any catalysts, additives, or metal oxides, and with excellent functional group compatibility. Control experiments revealed that water plays an essential role by bringing the reactive substrates into the aqueous medium where the intermediate polysulfide can form. Several examples of biologically active molecules such as thionicotinamide exhibiting anticancer activity have been obtained using this mild protocol. 

**Scheme 6:**
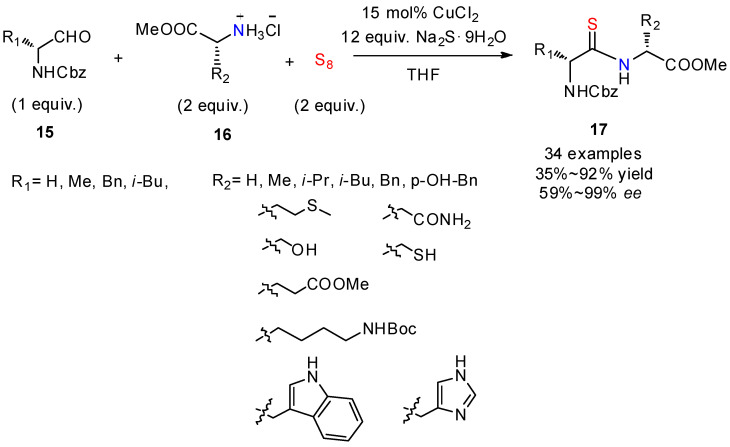
Cu-catalyzed synthesis of thioamide peptide by Jiang and co-workers [28].

The formation of thioamide peptides **17** by reaction of amino acids with amino aldehydes in the presence of S_8_ and sodium sulfide has been systematically explored by Jiang and co-workers (Scheme 6) [28]. The presence of copper II chloride appeared essential to the reaction by forming a N-Cu-N chelate fixing the imine, thus preventing racemization. A family of thioamide-containing analogs of dipeptides were prepared with high enantiomeric purity. A 15 mol% amount of CuCl_2_ was identified as the optimal catalyst amount to provide the desired products in good yields and with high enantioselectivity. This method provides a direct strategy for the synthesis of thioamide-containing peptides with chirality retention, and was efficiently applied to targets of pharmaceutical interest. Here, the presence of the catalyst is essential for preserving the chirality of the substrate into the product.

**Scheme 7:**
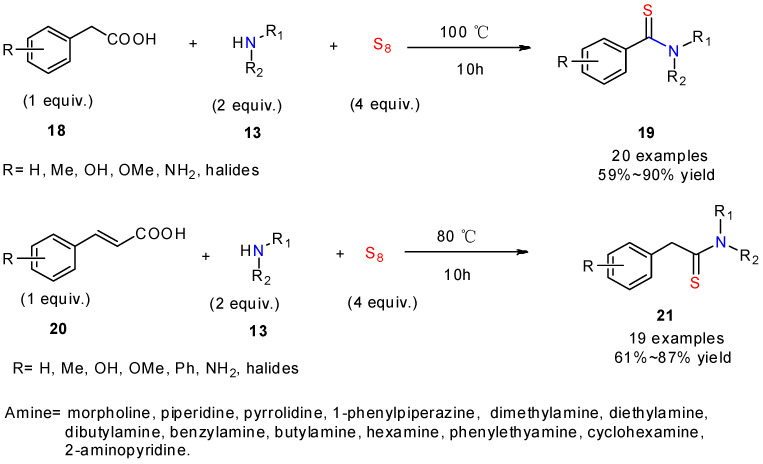
Decarboxylative reaction for the synthesis of thioamides by Singh and co-workers [29].

Decarboxylative reactions being a powerful strategy to construct the C-N bond, Singh’s group [29] reported a novel route towards benzothioamides **19** or 2-phenylethanethioamides **21** (Scheme 7). The reaction of aryl acetic or cinnamic acids with amines and elemental sulfur led to the formation of thioamides in the absence of any catalyst or solvent. The structural scope included various substituted arylacetic and cinnamic acids found to efficiently provide the target thioamides in good yields. In the case of nitro substituted substrates, the nitro group was reduced into an NH_2_ group that remained in the final product. This method, which proceeds without the use of catalyst or external oxidants, extended the scope of the Willgerodt–Kindler reaction to carboxylic acids, which are readily available starting materials.

**Scheme 8:**
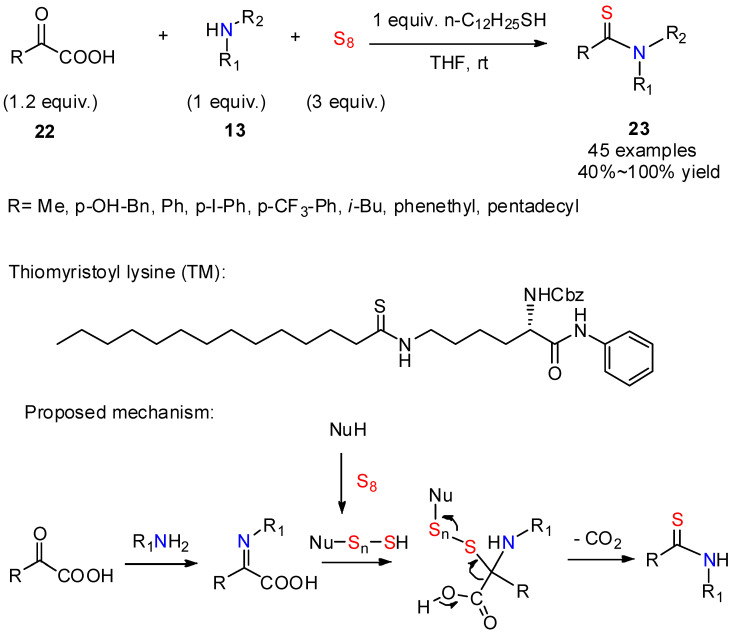
Thiol-mediated synthesis of aryl or alkyl thioamides by Takemoto’s group [30].

In 2020, Takemoto’s group [30] described a facile and site-selective approach to access thioamides using a α-keto carboxylic acid, amine, and sulfur via the nucleophilic addition of thiols to elemental sulfur (Scheme 8). The decarboxylative thioamidation between α-keto carboxylic acids **22** and amines **13** furnished the aryl/alkyl thioamides **23** in good yields. The reaction exhibited broad functional group tolerance, including unprotected alcohols, carboxylic acids, phenols, and unsaturated bonds. Examples of applications of the method included biologically active compounds such as thiomyristoyl lysine, possessing anticancer properties. The key to this approach is the activation of elemental sulfur by thiols. 

### 2.3. Thionylation of Cyanides, Halides, Azides, α-Nitroketones, Alcohols, Sulfoxonium Ylides, and Their Derivatives

**Scheme 9:**
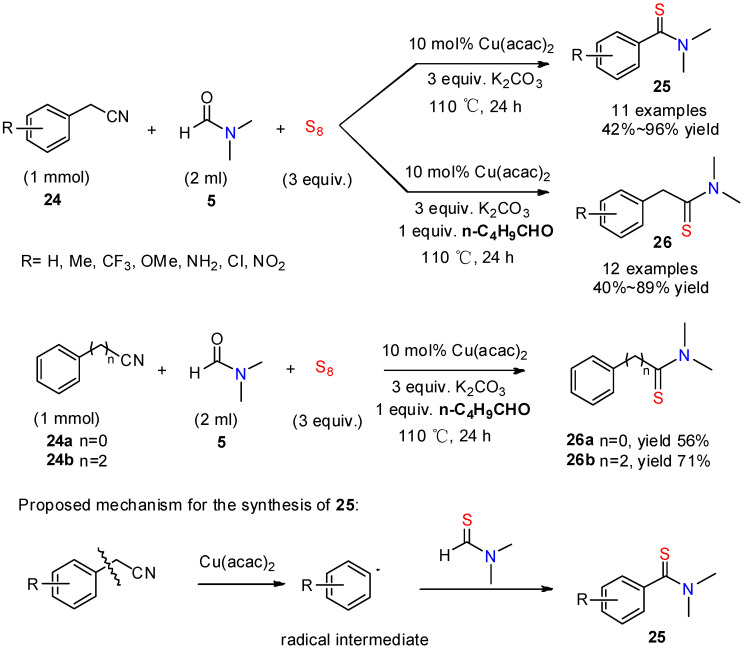
Cu-catalyzed synthesis of *N*,*N*-dimethylthioamides by Zhou and co-workers [31].

Copper-mediated selective thionylation of phenylacetonitrile with sulfur and dimethylformamide (DMF) was realized by Zhou’s group [31] to provide *N*,*N*-dimethylthiobenzamides **25** (Scheme 9). The exploration of the mechanism showed that the reaction proceeded via the formation of a benzene radical intermediate generated from Cu-catalyzed C-C bond cleavage of phenylacetonitrile and *N*,*N*-dimethyl sulfide amide generated from the reaction of elemental sulfur and DMF. When the reaction was performed in the presence of *n*-pentanal, *N*,*N*-dimethyl-2-phenylethanethioamides **26** were obtained (Scheme 9). Though the role of the aldehyde remained unclear, its ability to prevent the formation of benzene radicals was hypothesized. This method requires the presence of the copper catalyst to transform the initial phenylacetonitrile into the radical species.

**Scheme 10:**
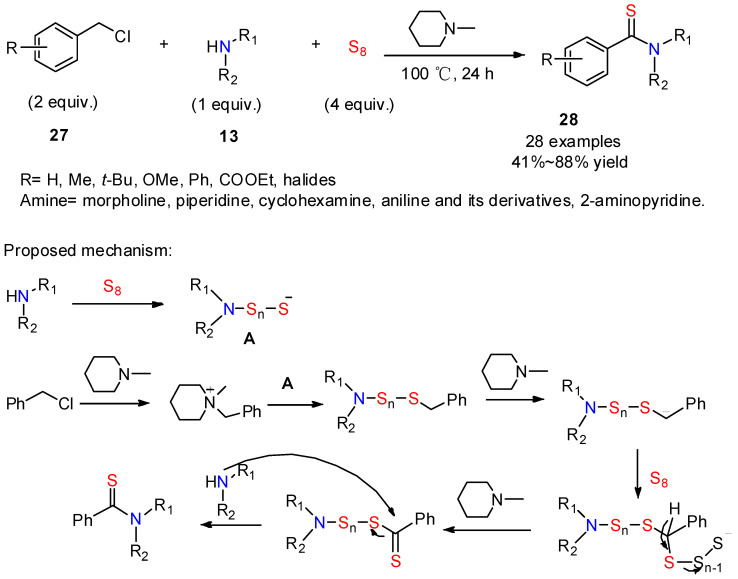
*N*-methylpiperidine-mediated synthesis of benzothioamides by Han and co-workers [32].

Benzyl chlorides can also lead to benzothioamides, thanks to a three-component reaction with amines and elemental sulfur, such as in the synthetic route established by Han and co-workers [32]. Under the optimized conditions, the reaction generated the desired benzothioamides **28** in yields up to 88% (Scheme 10). The scope of the reaction was investigated using a variety of amines and substituents on the aromatic ring of benzyl chloride. This is a very practical transformation which exhibits good functional group tolerance, and proceeds in a one-pot procedure without the utilization of transition metals or oxidants.

**Scheme 11:**
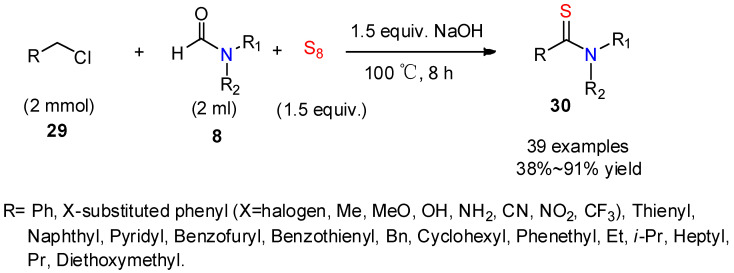
NaOH-mediated synthesis of thioamides by Zhou and co-workers [33].

Recently, another three-component thionylation involving aryl or alkyl chlorides, dimethylformamide, and elemental sulfur in a one-pot procedure was reported (Scheme 11) [33]. In this protocol, the starting dimethylformamide was not basic enough to activate the elemental sulfur, requiring addition of NaOH as a basic additive. Interestingly, this strategy extends the scope to alkyl chloride, while most other studies are limited to benzylic substrates.

**Scheme 12:**
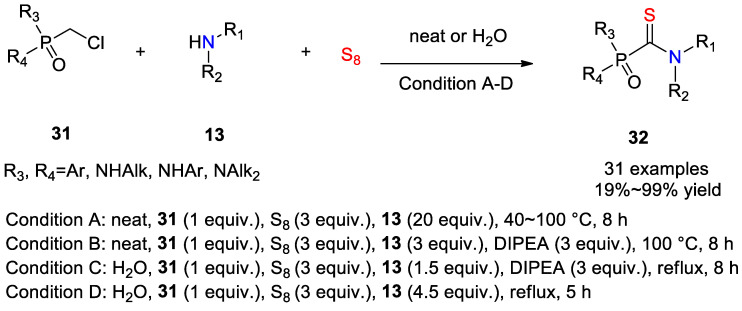
Synthesis of phosphoryl thioamides by Volkova and co-workers [34].

Phosphinic chlorides are interesting substrates towards phosphoryl thioamides and are precious intermediates in organophosphorus chemistry. Volkova’s group [34] developed an efficient, catalyst-free three-component preparation of phosphoryl thioamides **32** from phosphinic chlorides, amines, and elemental sulfur in water or under neat condition (Scheme 12). An excess of amines and sulfur was essential to provide the desired product in good yields, but the easy work-up allowed the complete recovery of excesses of substrates and reagents. Furthermore, the investigation of the substrate scope demonstrated the generality of the strategy.

**Scheme 13:**
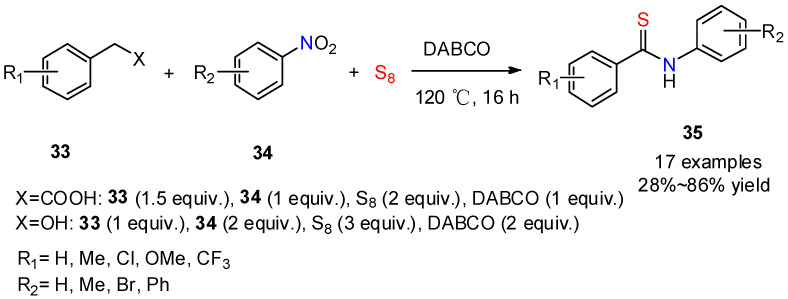
DABCO-mediated synthesis of benzothioamides by Phan and co-workers [35].

A three-component reaction to access *N*-phenyl-benzothioamides **35** involving benzyl alcohol or phenylacetic acid, nitroarenes, and elemental sulfur was developed in a one-pot procedure [35]. This method employed 1,4-diazabicyclo[2.2.2]octane (DABCO) as the base which activated the elemental sulfur (Scheme 13). The authors investigated the scope of substitution on the aryl acetic acid and nitroarene moieties, observing a good tolerance with respect to a wide range of functional groups. The extension of the reaction to benzyl alcohols is currently explored. Compared with other methods, the benefit of this approach is the use of nitroarenes as stable and readily available substrates.

**Scheme 14:**
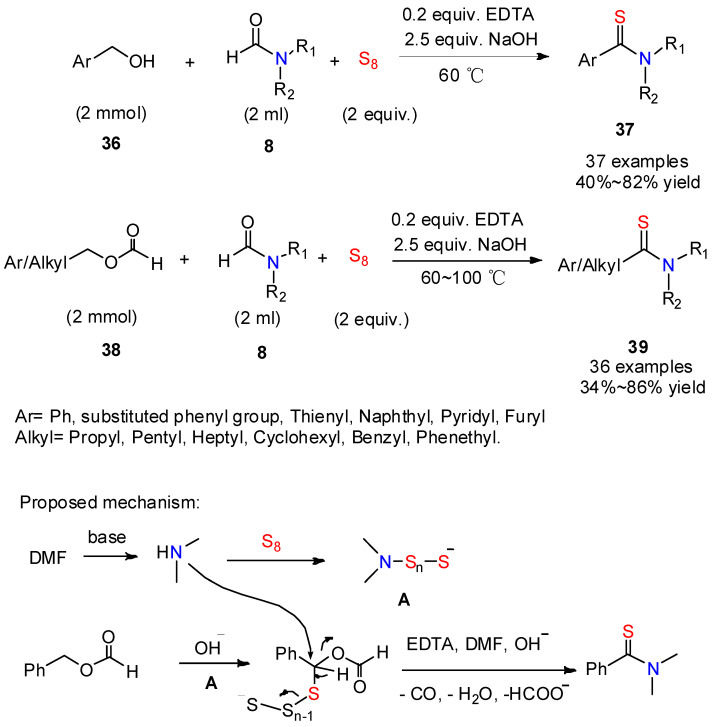
EDTA-mediated synthesis of aryl or alkyl thioamides by Zhou and co-workers [36].

More recently, a general strategy was developed for the one-pot synthesis of aryl or alkyl thioamide from variously substituted aryl methanol or their formate esters, in the presence of amides and elemental sulfur (Scheme 14) [36]. In this transformation, ethylene diamine tetraacetic acid (EDTA) served as a hydrogen delivery agent which transferred a proton from the benzylic carbon to a sulfur atom in the C-S coupling concerted C-H bond activation step. Alkyl alcohols were not able to undergo the same reaction because the hydroxyl group was preferably deprotonated in the initial C-H bond activation step. Benzyl formate being an intermediate in the formation of thioamides from benzylic alcohols, the authors found that alkyl formates were also able to provide the desired alkylthioamides. Formates thus appear as general substrates for thioamides synthesis.

**Scheme 15:**
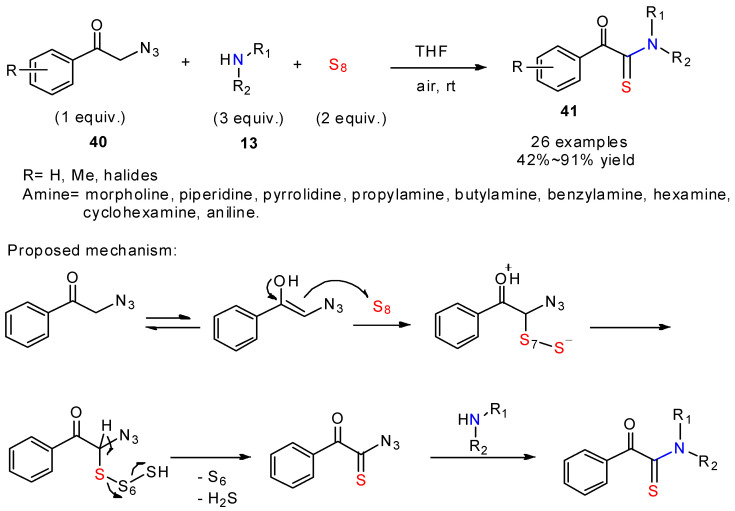
Oxidative amidation for the synthesis of α-keto thioamides by Chen and his colleagues [37].

Benefiting from the ability of azides to behave as leaving groups, Chen and his colleagues [37] described a metal-free synthetic approach towards α-ketothioamides **41** from α-azido ketones, amines, and elemental sulfur (Scheme 15). This reaction involving the cleavages of C-N_3_ displayed a broad substrate scope and high yields under mild conditions. On the basis of a series of control experiments, the authors proposed a plausible reaction mechanism via enol tautomerization, desulfhydrylation, and the departure of the azido group. This work provides a new development of α-azido ketone chemistry, and offers an alternative protocol for the synthesis of α-ketothioamide.

**Scheme 16:**
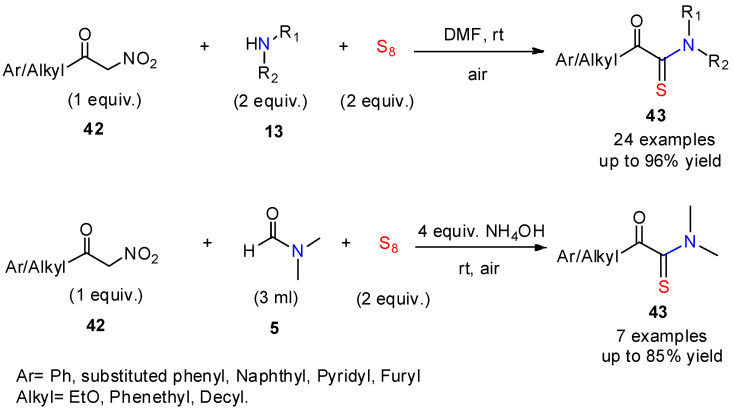
Oxidant-free synthesis of α-keto thioamides by Sheng and his colleagues [38].

α-Nitroketones are also useful substrates for the preparation of α-keto thioamides. In 2021, Sheng and his colleagues [38] described an efficient protocol for the synthesis of diverse α-keto thioamides **43** from α-nitroketones and amines in the presence of sulfur (Scheme 16). This transformation, which proceeds under mild condition, tolerates a broad range of substitutions, especially for the alkyl α-keto thioamides, which are rarely reported in the literature.

**Scheme 17:**
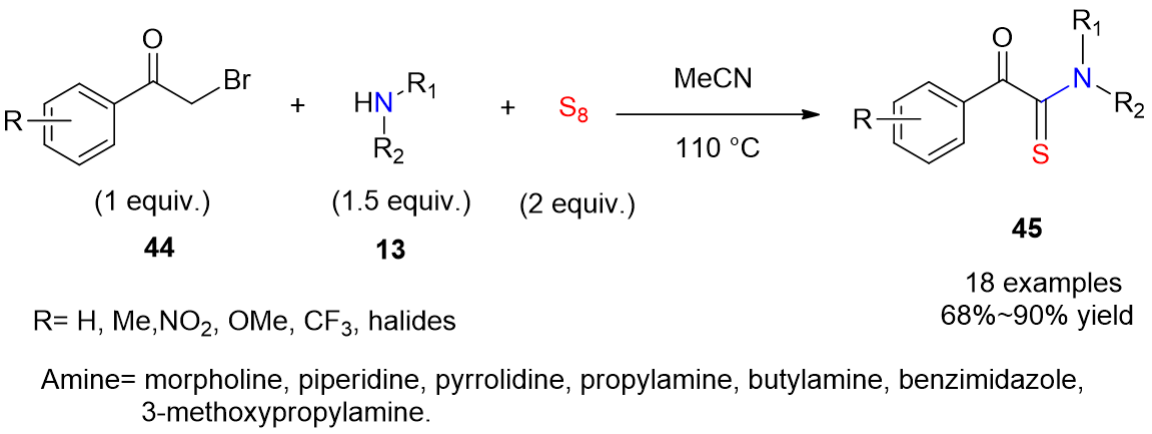
Synthesis of α-keto thioamides by Chen and co-workers [39].

In 2022, Chen’s group reported an efficient synthesis of α-keto thioamides via thiocarbonylation of C(sp^3^)-H of α-bromo ketones (Scheme 17) [39]. A wide range of α-bromo ketones and amines was well-tolerated in this transformation, leading to the formation of phenyl α-keto thioamides in good yields.

**Scheme 18:**
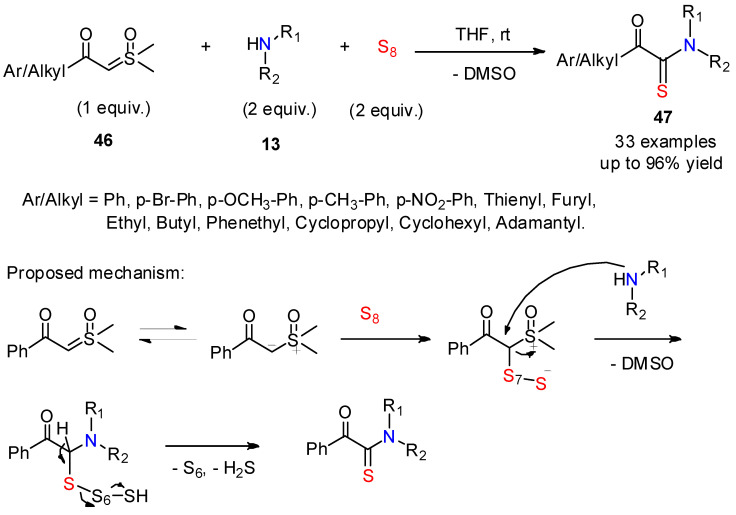
Synthesis of α-keto thioamides by Pandey and co-workers [40].

The same year, another access to α-keto thioamides was reported by Pandey’s group, who used sulfoxonium ylides as substrates with primary or secondary amines (Scheme 18) [40]. The reaction, driven by the release of DMSO, provided α-keto thioamides **47** at room temperature in good-to-excellent yields. This work provides an original development of the chemistry of sulfoxonium ylides, which are easy to synthesize and stock usually as stable crystalline solids. Using a ready operational procedure and mild conditions, this reaction leads to the formation of α-keto thioamides without the need of any catalysts or additional base.

### 2.4. Thionylation of Alkynes and Alkenes

**Scheme 19:**
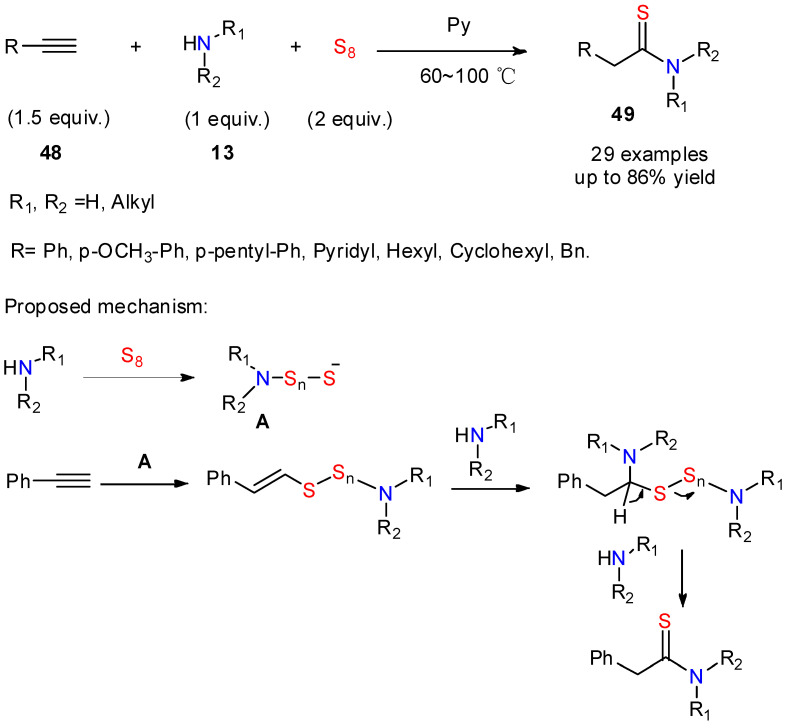
Synthesis of alkyl thioamides from alkynes by Nguyen and his colleagues [41].

Alkenes or alkynes can serve as substrates in thionylation reactions. For example, Nguyen and his colleagues [41] developed a straightforward approach to alkyl thioamides **49** through a three-component reaction from alkynes, aliphatic amines, and elemental sulfur (Scheme 19). The conversion of substrates was accelerated by the presence of pyridine making the reaction mixture more homogeneous. The reaction exhibits a remarkable tolerance for a wide range of functional groups and susbtituents on the alkyne and the alkylamine including cyclic secondary amines. Only aniline remained untransformed under these conditions. Mechanistic investigations led the authors to suggest that the reaction proceeded via the generation of a polysulfide **A**, its subsequent addition reaction onto the triple bond, then an addition reaction of the amino group onto the double bond, before final elimination of polysulfide. This work shows a remarkable example of atom- and step-economical reaction.

**Scheme 20:**
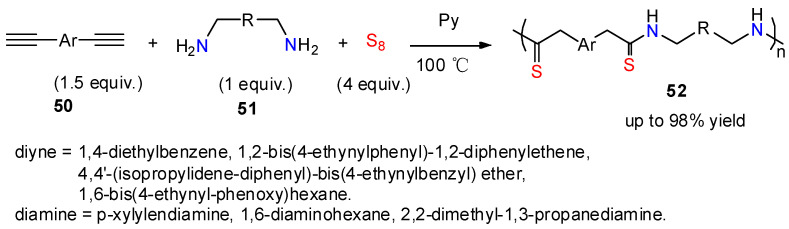
Synthesis of polythioamides by Tang and co-workers [42].

When diynes are used, the strategy can be applied to the preparation of polythioamides, which are interesting sulfur-containing polymers. Tang and his colleagues [42] employed aromatic diynes, elemental sulfur, and aliphatic diamines as monomers to synthesize the polythioamide **52** in high yield through multicomponent polymerization (Scheme 20). The resulting polymer exhibited high molecular weight and well-defined structure.

**Scheme 21:**
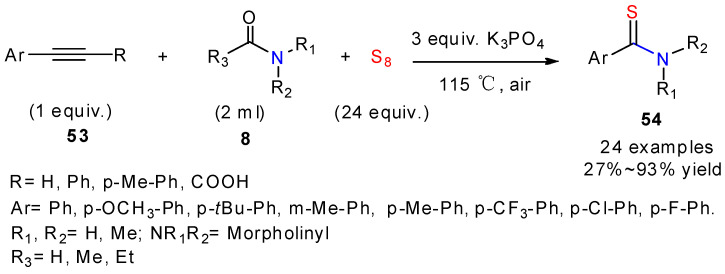
K_3_PO_4_-mediated synthesis of thioamides by Liu and co-workers [43].

In 2018, Liu and co-workers [43] reported the efficient three-component reaction of alkynes, amides, and elemental sulfur, which provided aryl thioamides such as **54** in moderate-to-good yields (Scheme 21). A first advantage of this protocol is to proceed in transition-metal-free conditions. Moreover, the cleavage of the C-C triple bond of aromatic alkynes can be promoted by elemental sulfur without any additional oxidants. This unexpected alkyne cleavage reaction was found to be of high tolerance with respect to the functional group substitution on the aryl alkynes.

**Scheme 22:**
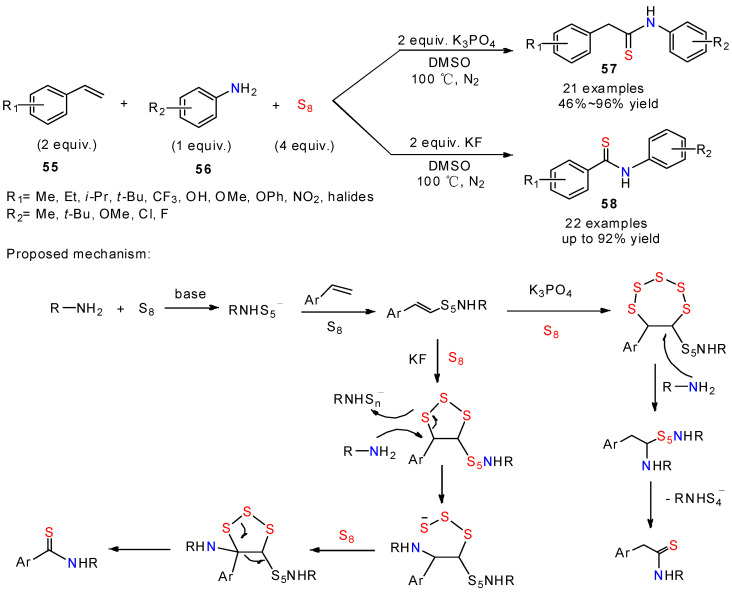
K_3_PO_4_-/KF-mediated synthesis of thioamides by Wu and co-workers [44].

A close example using alkenes was later described by Wu and his colleagues [44], who performed their three-component reaction with amines and sulfur in the presence of KF or K_3_PO_4_. Surprisingly, the two bases led chemoselectively to two different kinds of thioamides, the K_3_PO_4_-mediated reaction giving the 2-phenylethanethioamides **57** while the KF-mediated reaction provided benzothioamides **58**. Though still under investigation, the authors proposed a mechanism supported by control experiments involving the formation of the polysulfide RNHS_5_K (Scheme 22).

**Scheme 23:**
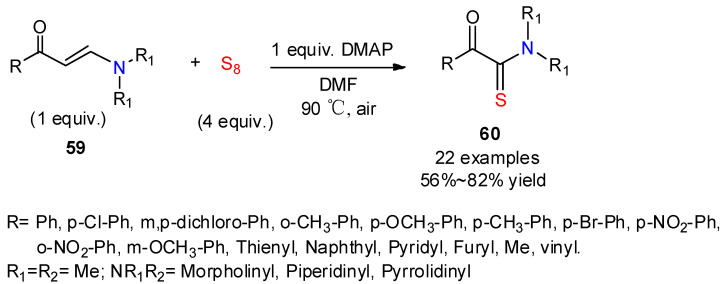
Synthesis of α-keto thioamides by Wan and co-workers [45].

The C=C bond of enaminoketones can also be involved in such a cleavage reaction. This was used by Wan and colleagues, who applied it to the synthesis of α-keto thioamides **60** in moderate-to-good yields by transformation of enaminoketones **59** in the presence of sulfur and DMAP under air (Scheme 23) [45]. This protocol provides an elegant and practical alternative for the construction of α-keto thioamides with broad substrate tolerance from easily available tertiary enaminones. The reaction failed to provide the desired product with *N*-methyl-*N*-phenyl tertiary enaminones, giving instead *N*,*N*-dimethyl α-keto thioamide, suggesting that DMF competed as an amido/amino source. Control experiments indicated that the cleavage of the C=C bond produced formaldehyde, which was further oxidized to formic acid.

**Scheme 24:**
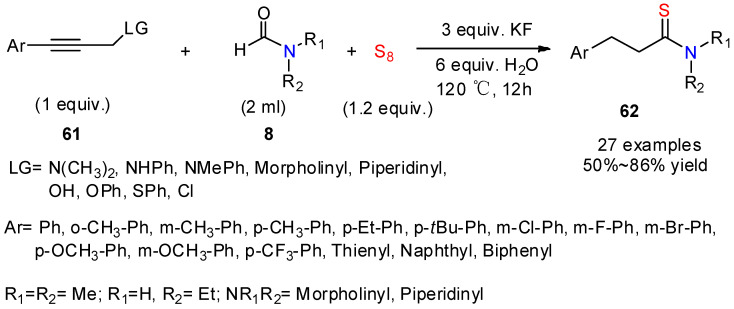
KF-mediated synthesis of aryl propanethioamides by Zhang and co-workers [46].

Formamides can also be used in place of amines, such as in the 2020 work reported by Zhang’s group [46] on the three-component reaction of arylpropynes **61**, formamides, and elemental sulfur (Scheme 24). The KF-mediated reaction led efficiently to the targeted aryl propanethioamides **62** with wide structural scope. The reaction mechanism was suggested to proceed first via hydrolysis of the C-C triple bond, to form the ketone, and concomitant hydrolysis of formamides, forming the secondary amines, and subsequent formation of the C-N bond and C-S double bond. Aryl propanethioamides **62** could be obtained from internal alkynes by this transformation, indicating that this process shared some similarities with the Willgerodt rearrangement.

**Scheme 25:**
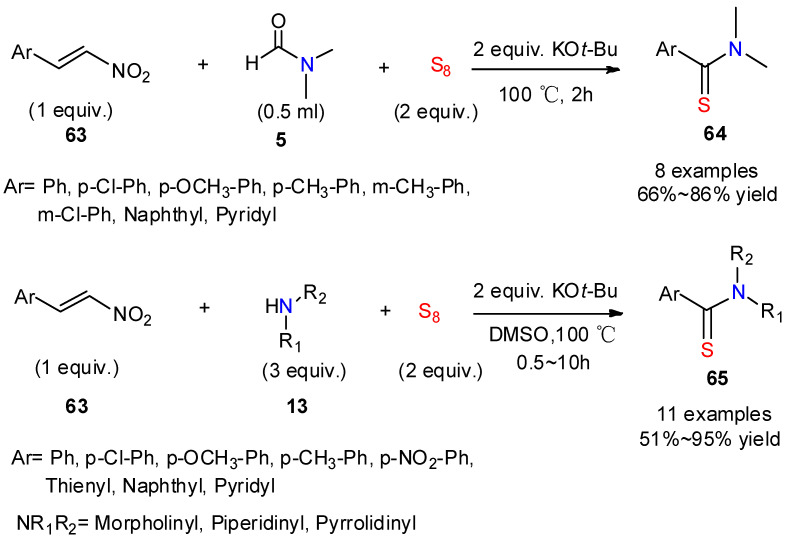
KO-*t*-Bu-mediated synthesis of aryl thioamides by Zeng and co-workers [47].

Alternatively, nitrostyrene can undergo a similar C=C bond cleavage process. In 2021, a three-component strategy towards aryl thioamides **64**/**65** was developed in Zheng’s group through a base-mediated C=C bond cleavage of β-nitrostyrene in the presence of sulfur and formamides or amines (Scheme 25) [47]. KO-*t*-Bu was found to be the most effective among organic or inorganic basic promoters of the reaction, leading to the corresponding thioamides in moderate-to-high yields.

**Scheme 26:**
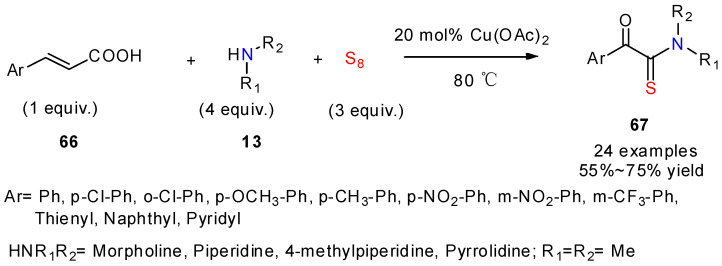
Cu-catalyzed synthesis of α-keto thioamides by Singh and co-workers [48].

Singh and co-workers reported in 2021 a very straightforward approach to construct α-keto thioamides, starting from readily available cinnamic acids and secondary amines (Scheme 26) relying on a copper-catalyzed decarboxylative thioamidation in the presence of sulfur under neat conditions [48]. α-Keto thioamides **67** were obtained as the final oxidative products in moderate yields. The presence of radical scavengers fully inhibited the formation of final oxidative products, suggesting that the reaction proceeded through a radical mechanism. Different from the one shown in Scheme 7, this reaction of cinnamic acids led to the formation α-keto thioamides. The presence of the copper catalyst is, however, indispensable, as it promotes the oxygenation step.

**Scheme 27:**
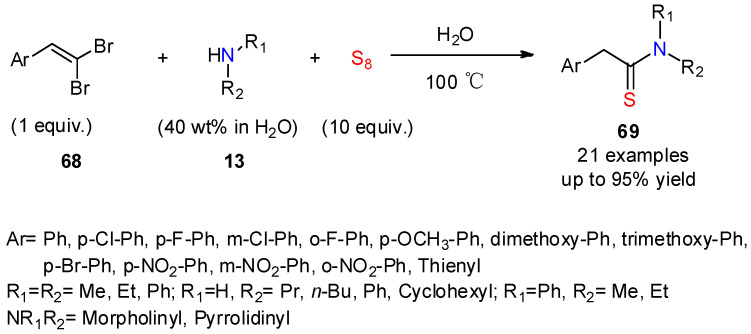
Synthesis of 2-arylethanethioamides in an aqueous medium by Gururaja and co-workers [49].

In 2021, Gururaja and co-workers reported the use of *gem*-dibromostyrenes as substrates in a catalyst-free, three-component reaction in aqueous medium leading to 2-arylethanethioamides **69** (Scheme 27) [49]. As in the case of aldehydes as substrates mentioned earlier (Scheme 5), water is essential for the thioamidation, which could be applied to a variety of aliphatic amines.

### 2.5. Thionylation of Methylheteroarenes

**Scheme 28:**
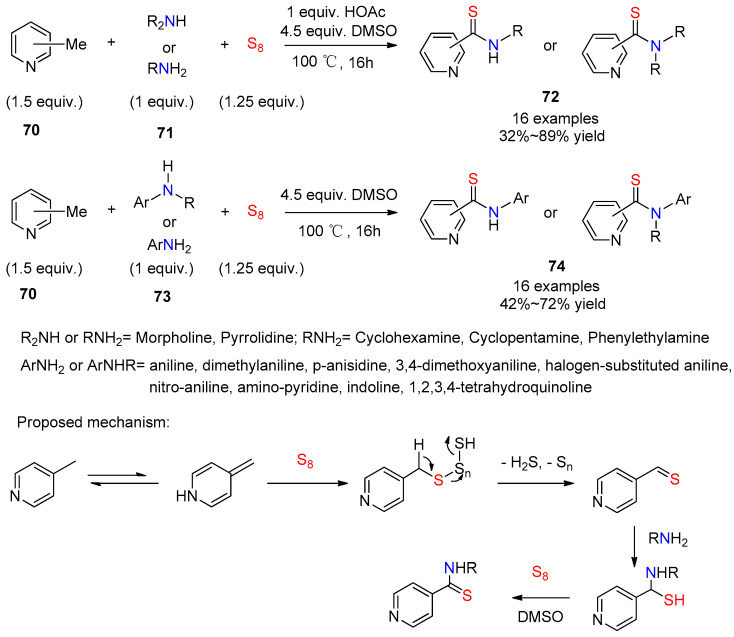
DMSO-promoted synthesis of aryl thioamides by Nguyen and co-workers [50].

Nguyen’s group [50] developed a thioamidation method via DMSO-promoted oxidative coupling of methylhetarenes with amines (Scheme 28). In their procedure, the loading of sulfur could be decreased as low as 1.25 equivalent, and a wide scope of substrates were tolerated to provide a variety of thioamides in high yield. Both DMSO and S_8_ were considered to act as oxidizing species. Addition of acetic acid was required for the reaction of aliphatic amines, which gave the expected thioamides **72**, and aromatic amines could be transformed to the desired products **74** in high yields even without any additive. Compared with the protocols without DMSO, this protocol is advantageous by decreasing the reaction temperature and the load of sulfur.

### 2.6. Thionylation Involving Amines Only

**Scheme 29:**
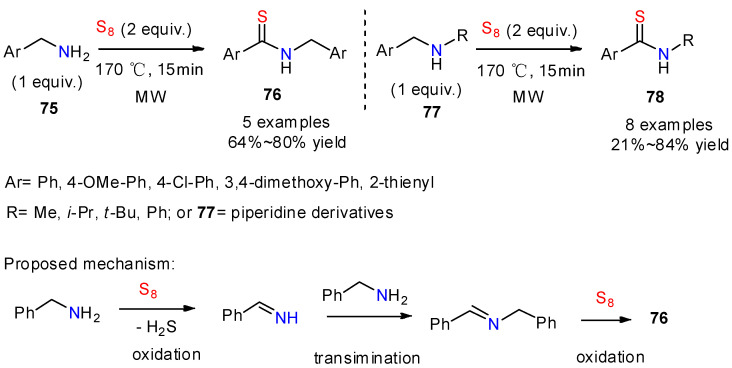
Microwave-assisted synthesis of aromatic thioamides by Keglevich and co-workers [51].

In the absence of any other partner, primary or secondary amines can be transformed to thioamides by reaction with S_8_. For example, under microwave irradiation and in solvent-free conditions, variously substituted benzylamines **75** or **77** could be converted in moderate-to-good yields to the aromatic thioamides **76** or **78**, respectively (Scheme 29) [51]. The main benefit of this method is the by far shorter reaction time compared to previously reported methods. The generality of the reaction needs further investigation for its extension to secondary amines, as only low yields were observed for some examples such as *N*-tert-butyl and *N*-phenylbenzylamines.

**Scheme 30:**
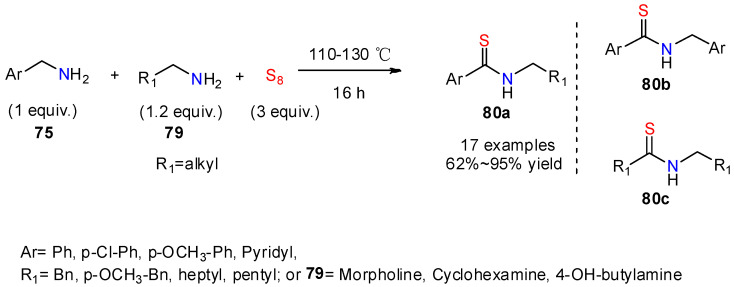
Oxidative coupling of two amines into aromatic thioamides by Nguyen and his colleagues [52].

If two different primary amines are mixed together in the presence of elemental sulfur, four possible thioamides can generally be formed in the reaction. Nguyen and his colleagues found, however, that cross-coupling products could be obtained with significant selectivity because benzyl amines are more prone to oxidation [52]. They reported the preparation of a series of cross-coupled thioamides, indicating that only one heterocoupled thioamide **80a** was the major product, while only small amounts of homocoupled thioamides **80b** and **80c** were observed as by-products (Scheme 30).

**Scheme 31:**
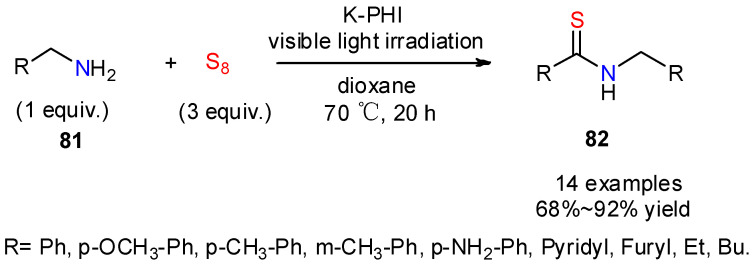
Photocatalytic synthesis of aryl/alkyl thioamides by Savateev and co-workers [53].

The formation of thioamides from amines and elemental sulfur can be photocatalyzed. A recent example is the work of Savateev’s group in 2018 [53]. They reported the synthesis of aryl or alkyl thioamides **82** under visible light irradiation in the presence of potassium poly(heptazine imide) (K-PHI) as a photocatalyst (Scheme 31). In terms of scope, a wide range of substituted benzylamines and heterocyclic or aliphatic methylamines were transformed to thioamides in high isolated yields, except for 2-methyl benzylamine, which was unreactive under these conditions. The authors suggested that steric hindrance of the methyl group near the reactive site was responsible for this lack of reactivity.

**Scheme 32:**
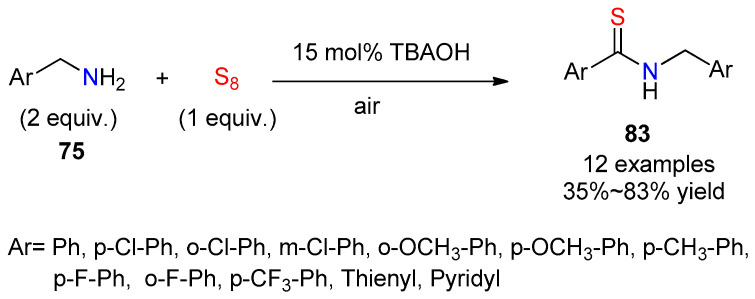
Ionic liquid TBAOH catalyzed synthesis of *N*-benzylbenzothioamides by Adimurthy and co-workers [54].

Catalysis of the thioamidation can also come from the addition of tetrabutylammonium hydroxide (TBAOH), as reported by Adimurthy and his colleagues, who described the transformation of benzyl amines to *N*-benzylbenzothioamides **83** with elemental sulfur under aerobic conditions (Scheme 32) [54]. The corresponding thioamides were provided in good yields using different benzyl amines with electron-withdrawing or electron-donating groups. Interestingly, 2-chlorobenzylamine could be transformed to the corresponding thioamide under these conditions, despite the steric hindrance of the chloride, which normally limits its reactivity. In this formation, ionic liquid TBAOH acted as catalyst and aerobic oxygen as a sole oxidant under solvent- and base-free conditions. The authors further expanded the scope of their study to heterothioamides.

**Scheme 33:**
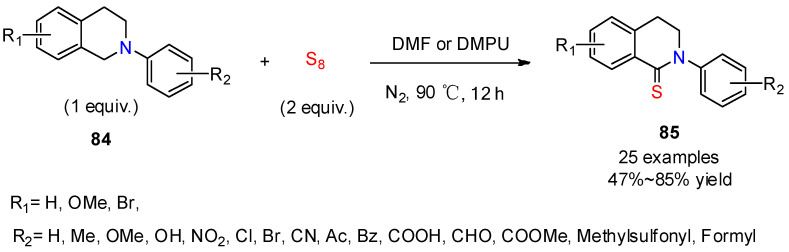
C(sp^3^)-H thiocarbonylation for thioamides by Wei and his co-workers [55].

A solvent-driven C(sp^3^)-H thiocarbonylation of bicyclic benzylic amines such as **84** into 2-phenyl-3,4-dihydroisoquinoline-1(2*H*)-thione derivatives **85** was developed by Wei and co-workers (Scheme 33) [55]. Conducted under catalyst-free conditions, the reaction was specific to dipolar aprotic solvents such as DMF, DMA, and DMPU. Mechanistic calculations of the complete reaction free energy profiles for the different benzylamines in DMPU as solvent led to the proposal that the reaction proceeded via the formation of S_3_^•−^ species and the solvent radical anion (DMPU^•−^).

## 3. Inorganic Sulfides as a Sulfuration Agent

In this section, we review how inorganic sulfides such as sodium hydrosulfide (NaSH), sodium sulfide (Na_2_S), and sodium disulfide (Na_2_S_2_) have been recently used as sulfur sources in strategies targeting thioamides. Using inorganic sulfide is indeed an attractive source of sulfur for organic reactions with numerous occurrences and applications in industrial processes and material science.

**Figure 4:**
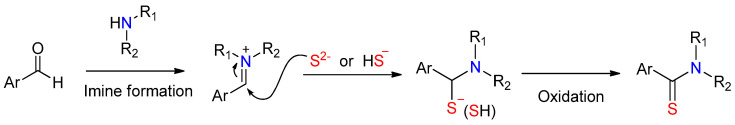
The mechanism of the inorganic sulfur-mediated thioamide synthesis.

Though not as convenient as compared to elemental sulfur because some hydrogen sulfide is generally released, inorganic sulfides are, however, useful reagents, being still preferable compared to organosulfur reagents or Lawesson and P_4_S_10_ reagents in terms of cleanness and practicality, while covering a wide range of possible substrates. These practical aspects have confirmed them as useful reagents in organic synthesis. Some recent efforts to improve the cleanness and practicality of these latter methods are briefly discussed below. Mechanistically, in the case of aldehydes, the reaction process involves the formation of an imine, which undergoes sulfur anion or HS- addition before the final oxidation step (Figure 4).

### 3.1. Sodium Hydrosulfide as a Sulfuration Agent

Sodium hydrosulfide is a water-soluble and stable salt. When dissolved in water, a highly alkaline sodium hydrosulfide (pH range of 11.5 to 12.5) is obtained. NaSH is not very cheap, but it can be readily prepared by reacting hydrogen sulfide with sodium ethoxide in ethanol.

**Scheme 34:**
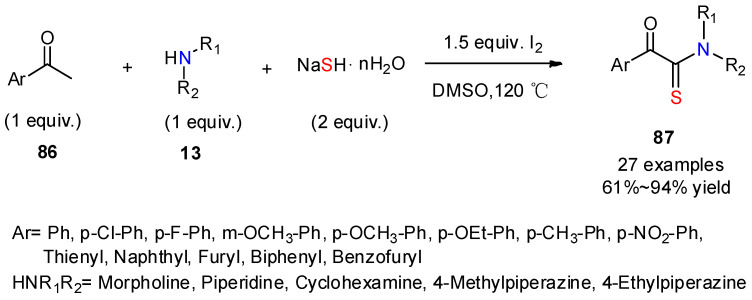
I_2_-mediated synthesis of α-keto thioamides by Wu and co-workers [56].

A recent study by Wu’s group focused on the use of sodium hydrosulfide as a sulfur source in a three-component reaction starting from amines and aryl methyl ketones promoted by iodine (Scheme 34) [56]. This method allowed the direct and efficient conversion of aryl methyl ketones to α-keto thioamides **87**, which were not able to be directly prepared through the original Willgerodt–Kindler reaction from acetophenone. The optimal reaction conditions (1.5 equiv I_2_ at 120 °C in DMSO) were applied to the reaction of acetophenone with a variety of secondary amines, leading smoothly to the corresponding α-ketothioamides in good yields. In this process, iodine played the role of an oxidant which could be regenerated from the Kornblum oxidation of the byproduct HI in the presence of DMSO.

**Scheme 35:**
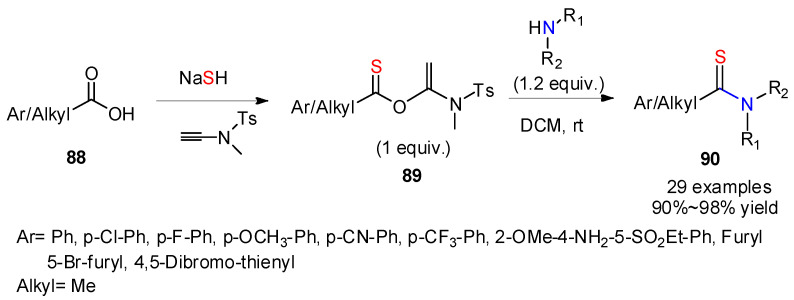
Ynamide-mediated synthesis of thioamides by Zhao and co-workers [57].

NaSH served as the sulfur source in another approach converting carboxylic acids to thioamides. In this method, the carboxylic acids were first transformed into corresponding thiocarboxylic acids in the presence of NaSH. A second step is the addition of the thiocarboxylic acids on the ynamide (*N*-methylynetoluenesulfonamide MYTsA), forming an intermediate thioester **89** (Scheme 35). The thioamidation then occurs by reaction of this latter with amines, leading to aryl or alkyl thioamides **90** in excellent yields and wide functional group tolerance [57]. Thanks to the mildness of the conditions avoiding any epimerization or racemization of chiral substrates, this method has found remarkable applications in the synthesis of thiopeptides [58].

### 3.2. Sodium Sulfide as a Sulfuration Agent

Sodium sulfide, often used as its hydrate, is a water-soluble salt that is readily available and very cheap. Sodium sulfide is stable in basic conditions. 

**Scheme 36:**
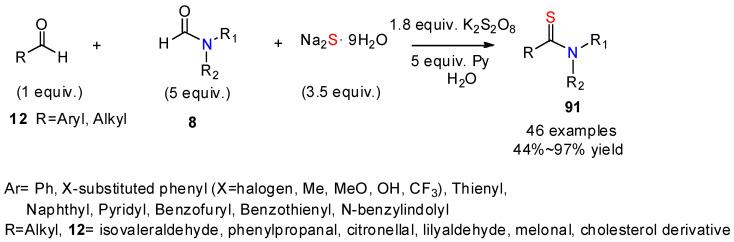
Na_2_S-mediated synthesis of aryl or alkyl thioamides in water by Jiang and co-workers [59].

Jiang’s group [59] reported an efficient and practical three-component preparation of aryl/alkyl thioamides **91** in water involving aryl or alkyl aldehydes, *N*-substituted formamides, and sodium sulfide (Scheme 36). *N*-substituted formamides are essential for this transformation. Comparatively, the corresponding amines led to a trace amount of the targeted thioamides, owing to the hydrolysis of formamide by sodium sulfide to release hydrogen sulfide, acting as a promoter of the reaction. This protocol proved to tolerate several functional groups, notably for aryl aldehydes and unsaturated aliphatic aldehydes. This is an important advantage of this method, which could be applied to late-stage modifications of bioactive drugs containing carbon-carbon double bonds.

**Scheme 37:**
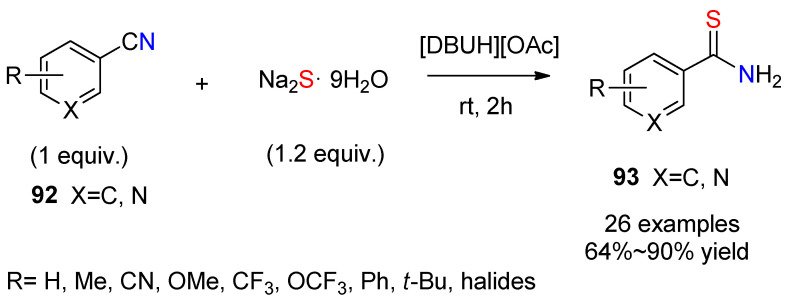
Ionic liquid [DBUH][OAc]-promoted synthesis of thioamides by Zhang and co-workers [60].

It is worth mentioning another example of sodium sulfide-mediated thionylation which benefited from the use of an ionic liquid as medium. This work concerned the formation of thiobenzamide derivatives **93** from aryl nitriles promoted by [DBUH][OAc] ionic liquid at room temperature (Scheme 37) [60]. This was well-applicable to a wide range of functional aryl nitriles. The ionic liquid allowed easy purification from the reaction mixture of the target products and could be reused several times (same catalytic ability even five rounds).

**Scheme 38:**
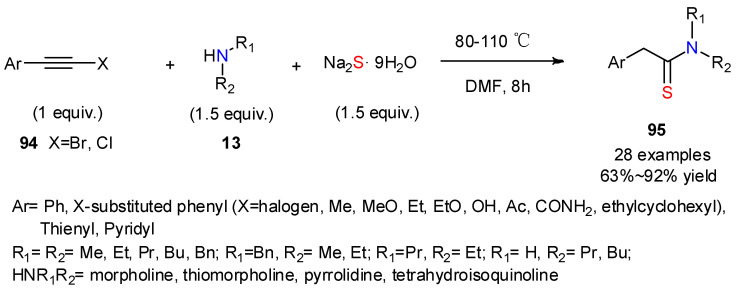
Na_2_S-mediated synthesis of 2-arylethanethioamides in DMF by Jiang and co-workers [61].

A recent example of thionylation using sodium sulfide as a source of sulfur was reported by Sun and co-workers for the transformation of 2-aryl haloalkynes into 2-arylethanethioamides (Scheme 38) [61]. They established an efficient three-component reaction under rather mild conditions using DMF as the best solvent among several others, such as 1,4-dioxane, and DME. A wide range of amines and different functional haloalkynes were examined to afford the expected thioamides **95** in good yield. The proposed mechanism based on isotopic exchange control experiments showed that, among the two hydrogen atoms of the methylene group in **95**, one arose from the amine, and one from water, indicating that the presence of the water molecules (arising from Na_2_S·9H_2_O) is essential.

**Scheme 39:**
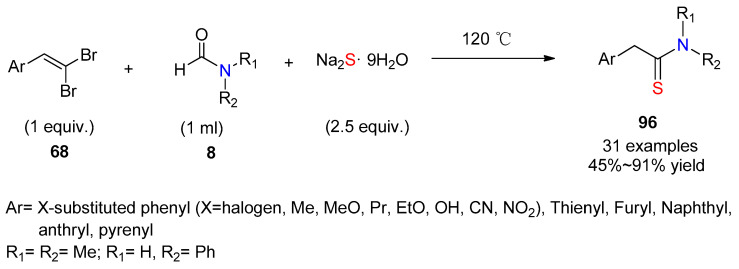
Na_2_S-mediated synthesis of 2-arylethanethioamides by Doddi and co-workers [62].

Doddi and co-workers used sodium sulfide and *N*-substituted formamide in a three-component transformation of *gem*-dibromostyrenes (Scheme 39) [62]. A series of 2-arylethanethioamides **96** were obtained in fair-to-excellent yields in the absence of any catalyst. The authors indicated that the transformation starts by the formation of an intermediate alkyne.

### 3.3. Sodium Disulfide as a Sulfuration Agent

**Scheme 40:**
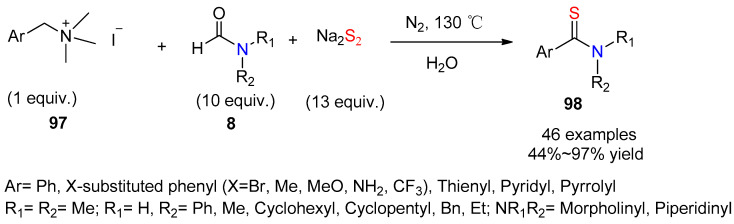
Na_2_S_2_-mediated synthesis of aryl thioamides by Cheng and co-workers [63].

Sodium disulfide is a water-soluble yellow salt. Sodium disulfide is not stable and causes the release of H_2_S in the air and light. Though unstable, it can be a useful sulfur source for thionylation reactions, such as in the transformation of quaternary ammonium salts and amides reported recently by Cheng and his colleagues [63]. They developed a catalyst-free protocol towards aryl thioamides **98** involving aryl trimethyl ammonium iodide, *N*-substituted formamides, and aqueous sodium disulfide (Scheme 40). This transformation gave the expected thioamides in moderate-to-good yields with high functional group tolerance. In their study, several inorganic sulfur sources were compared, including elemental sulfur, sodium sulfide, potassium sulfide, and sodium disulfide. The advantage of sodium disulfide was to provide good results without additional oxidant required.

## 4. Recent Practical Improvements of Other Thionylation Methods

Though less attractive with respect to cleanness and toxicity, we want to mention some efforts which have been made for improving other traditional methods, namely, the use of P_4_S_10_ or Lawesson’s reagent, and reactions of organosulfur compounds, as these methods offer in some cases a shorter option with respect to the access to the substrate itself. For example, as the LR is inefficient at relatively high temperatures due to its rapid decomposition, Svensson and co-workers [64] used P_4_S_10_ in pyridine as a thionating agent in DMSO at high temperatures. In the workup procedure, the remaining thionating reagent can be transferred to solvents by addition of water and the target products can be easily obtained with high purity by recrystallization. Organosulfur is also a well-documented source of sulfur to construct thioamides, especially thiols [65], disulfides [66,67,68], and thiourea [69], primary or secondary thioamides [70,71], and 1,2,3-thiadiazoles [72]. However, organosulfur compounds are featured as catalyst poisons for transition metals due to their ability to coordinate metallic species [73], which sometimes limits their application. Overcoming these drawbacks has thus been an aim, as in some protocols based on transition-metal-free transamidation reactions, operationally simple and broadly functional group-compatible [70,71].

**Scheme 41:**
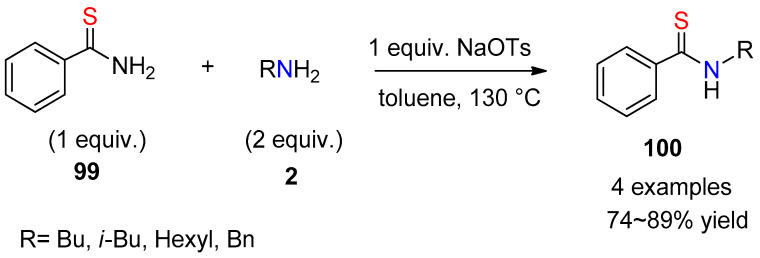
Transamidation of aryl thioamides by Zeng and co-workers [70].

The transamidation reaction of thioamides has also been recently considered as a sensible solution for the synthesis of substituted thioamides. In 2021, Zeng and co-workers [70] applied NaOTs as promoter to activate unsubstituted thioamides towards new C-N bond formation (Scheme 41), which provides a simple pathway to access substituted thioamides.

**Scheme 42:**
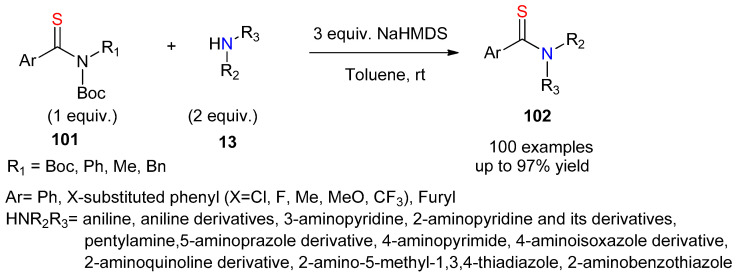
Transamidation of aryl thioamides by Szostak and co-workers [71].

In the same trend, Szostak and co-workers reported in 2022 a simple and direct method for transamidation of *N*-tert-butoxycarbonyl aryl thioamides **101** based on the uncommon cleavage of the C(S)-N bond under mild conditions [71]. This process occurred well in the presence of NaHMDS, whereas other bases (NaOH, KO-*t*-Bu, *n*-BuLi) were found ineffective in this reaction (Scheme 42). This protocol is broadly compatible with different functional groups, thus offering an interesting alternative for the construction of substituted thioamides.

**Scheme 43:**
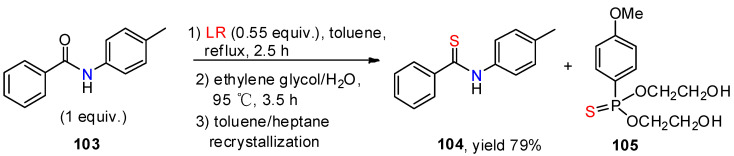
Thionation reactions with LR by Hu and co-workers [74].

Regarding P_4_S_10_ and Lawesson’s reagent, which often produce toxic phosphor-containing aqueous chemical wastes and require tedious procedures, some improved work-ups have been proposed. Recently, Hu and co-workers [74] developed an efficient work-up for their thionation reactions with LR by a treatment of ethylene glycol (Scheme 43) which avoided purification by column chromatography and the generation of phosphor-containing aqueous waste.

## 5. Conclusions

While advanced chemical synthesis techniques for directly preparing thioamide moieties have become common, there is an increasing interest in efficient and more practical methods applied to thioamide-containing natural products. However, sulfur incorporation in organic molecules remains a challenge from the practical viewpoint. In this review, we have tried to highlight some recent studies which all try to improve the feasibility of the preparation of thioamides, which are very important targets in organic and medicinal chemistry. Focusing first on elemental sulfur, which is by far the most practical and clean reagent, we have highlighted numerous very useful methods which continue to widen the scope of substrates, solvents, catalysts, and the remarkably wide scope of possible targets.

Many protocols performed in water or solvent-free conditions provide various types of thioamides in high yields, which makes their process clean and significantly improved in the context of more environmentally friendly chemical transformations. The transamidation of thioamides appears also as a sensible alternative as it is operationally easy, user-friendly, and conducted under rather mild conditions, though necessitating the addition of base. The ability of a transformation to be performed on a very complex substrate is of high importance. Thus, the processes which exhibit wide functional group tolerance are the most attractive ones as they can be applied to the late-stage functionalization of drug candidates. Important also is the ability to preserve chirality of chiral substrates, and in this regard, the presence of catalysts remains often essential.

These methods, more practical and already quite general, can though still be improved with respect to the amine substrate scope, some of them such as aliphatic primary amines remaining more challenging. Other methods, yet not as ideal as compared to elemental sulfur, have also been mentioned when some kind of improvement is proposed. Overall, these new protocols and methods open up new chances to discover unconventional reactions for thioamides and their derivatives.

## Data Availability

See bibliographic data cited in the References section below.

## References

[1] Mueller E.G. (2006). Trafficking in persulfides: Delivering sulfur in biosynthetic pathways. Nat. Chem. Biol..

[2] Schwalen C.J., Hudson G.A., Kille B., Mitchell D.A. (2018). Bioinformatic expansion and discovery of thiopeptide antibiotics. J. Am. Chem. Soc..

[3] Kenney G.E., Dassama L.M., Pandelia M.-E., Gizzi A.S., Martinie R.J., Gao P., DeHart C.J., Schachner L.F., Skinner O.S., Ro S.Y. (2018). The biosynthesis of methanobactin. Science.

[4] Hooper M. (1987). The medicinal chemistry of anti-leprosy drugs. Chem. Soc. Rev..

[5] Lincke T., Behnken S., Ishida K., Roth M., Hertweck C. (2010). Closthioamide: An unprecedented polythioamide antibiotic from the strictly anaerobic bacterium *Clostridium cellulolyticum*. Angew. Chem. Int. Ed..

[6] Banala S., Süssmuth R.D. (2010). Thioamides in nature: In search of secondary metabolites in anaerobic microorganisms. ChemBioChem.

[7] Mahanta N., Szantai-Kis D.M., Petersson E.J., Mitchell D.A. (2019). Biosynthesis and chemical applications of thioamides. ACS Chem. Biol..

[8] Litomska A., Ishida K., Dunbar K.L., Boettger M., Coyne S., Hertweck C. (2018). Enzymatic thioamide formation in a bacterial antimetabolite pathway. Angew. Chem. Int. Ed..

[9] Chatterjee S., Hausinger R.P. (2022). Sulfur incorporation into biomolecules: Recent advances. Crit. Rev. Biochem. Mol. Biol..

[10] Kumari S., Carmona A.V., Tiwari A.K., Trippier P.C. (2020). Amide bond bioisosteres: Strategies, synthesis, and successes. J. Med. Chem..

[11] Lampkin B.J., VanVeller B. (2021). Hydrogen bond and geometry effects of thioamide backbone modifications. J. Org. Chem..

[12] Fiore K.E., Patist M.J., Giannakoulias S., Huang C.H., Verma H., Khatri B., Cheng R.P., Chatterjee J., Petersson E.J. (2022). Structural impact of thioamide incorporation into a β-hairpin. RSC Chem. Biol..

[13] Jagodziñski T.S. (2003). Thioamides as useful synthons in the synthesis of heterocycles. Chem. Rev..

[14] Gasteiger J., Herzig C. (1981). Synthetic applications of 2-chlorooxiranes: Preparation of thiazoles, dihydrothiazoles and selenazoles. Tetrahedron.

[15] Singh H., Sarin R. (1986). Carbon transfer reactions of Δ^2^-oxazolinium and thiazolinium cations. Tetrahedron.

[16] Engman L. (1991). Organoselenium- and proton-mediated cyclization reactions of allylic amides and thioamides. Syntheses of 2-oxazolines and 2-thiazolines. J. Org. Chem..

[17] Joyce L.L., Evindar G., Batey R.A. (2004). Copper- and palladium-catalyzed intramolecular C–S bond formation: A convenient synthesis of 2-aminobenzothiazoles. Chem. Commun..

[18] Cheng Y., Peng Q., Fan W., Li P. (2014). Room-temperature ligand-free Pd/C-catalyzed C–S bond formation: Synthesis of 2-substituted benzothiazoles. J. Org. Chem..

[19] Ozturk T., Ertas E., Mert O. (2010). A berzelius reagent, phosphorus decasulfide (P_4_S_10_) in organic syntheses. Chem. Rev..

[20] Ozturk T., Ertas E., Mert O. (2007). Use of Lawesson’s reagent in organic syntheses. Chem. Rev..

[21] Priebbenow D.L., Bolm C. (2013). Recent advances in the Willgerodt-Kindler reaction. Chem. Soc. Rev..

[22] Nguyen T.B. (2017). Recent advances in organic reactions involving elemental sulfur. Adv. Synth. Catal..

[23] Xu H., Deng H., Li Z., Xiang H., Zhou X. (2013). Synthesis of thioamides by catalyst-free three-component reactions in water. Eur. J. Org. Chem..

[24] Liu W., Chen C., Liu H. (2015). Dimethylamine as the key intermediate generated in situ from dimethylformamide (DMF) for the synthesis of thioamides. Beilstein J. Org. Chem..

[25] Li J., Ren X., Li G., Liang H., Zhao Y., Wang Z., Li H., Yuan B. (2020). Mixed bases mediated synthesis of thioamides in water. J. Sulfur. Chem..

[26] Kale A.D., Dalal D.S. (2022). Catalyst- and solvent-free thioamidation of aromatic aldehydes through a Willgerodt-Kindler reaction. ChemistrySelect.

[27] Gupta A., Vankar J.K., Jadav J.P., Gururaja G.N. (2022). Water mediated direct thioamidation of aldehydes at room temperature. J. Org. Chem..

[28] Liao Y., Jiang X. (2021). Construction of thioamide peptide via sulfur-involved amino acids/amino aldehydes coupling. Org. Lett..

[29] Guntreddi T., Vanjari R., Singh K.N. (2014). Decarboxylative thioamidation of arylacetic and cinnamic acids: A new approach to thioamides. Org. Lett..

[30] Saito M., Murakami S., Nanjo T., Kobayashi Y., Takemoto Y. (2020). Mild and chemoselective thioacylation of amines enabled by the nucleophilic activation of elemental sulfur. J. Am. Chem. Soc..

[31] Qu Y., Li Z., Xiang H., Zhou X. (2013). Copper (II)-catalyzed reactions of dimethylformamide with phenylacetonitrile and sulfur to form *N*,*N*-dimethylthioamides. Adv. Synth. Catal..

[32] Li X., Pan Q., Hu R., Wang X., Yang Z., Han S. (2016). S_8_-mediated one-pot synthesis of thioamides from benzyl chlorides and amines. Asian J. Org. Chem..

[33] Jin H., Chen X., Qian C., Ge X., Zhou S. (2021). Transition-metal-free, general construction of thioamides from chlorohydrocarbon, amide and elemental sulfur. Eur. J. Org. Chem..

[34] Kozlov M., Kozlov A., Komkov A., Lyssenko K., Zavarzin I., Volkova Y. (2019). Synthesis of phosphoryl thioamides via three-component reaction of phosphinic chlorides with amines and sulfur. Adv. Synth. Catal..

[35] Do N.T., Tran K.M., Phan H.T., To T.A., Nguyen T.T., Phan N.T. (2019). Functionalization of activated methylene C–H bonds with nitroarenes and sulfur for the synthesis of thioamides. Org. Biomol. Chem..

[36] Jin H., Ge X., Zhou S. (2021). General construction of thioamides under mild conditions: A stepwise proton transfer process mediated by EDTA. Eur. J. Org. Chem..

[37] Yu P., Wang Y., Zeng Z., Chen Y. (2019). Metal-free C–N or C–C bond cleavages of α-azido ketones: An oxidative-amidation strategy for the synthesis of α-ketothioamides and amides. J. Org. Chem..

[38] Zhang Z., Yang J., Yu R., Wu K., Bu J., Li S., Qian P., Sheng L. (2021). Efficient synthesis of α-ketothioamides from α-nitroketones, amines or DMF and elemental sulfur under oxidant-free conditions. Eur. J. Org. Chem..

[39] Tian H., Guo F., Chen X. (2022). C*_sp3_*-H bond functionalization of α-bromo ketones for the synthesis of α-keto thioamides using elemental sulfur. Russ. J. Org. Chem..

[40] Chaubey T.N., Borpatra P.J., Sharma A., Pandey S.K. (2022). Metal-free syntheses of α-keto thioamide and α-ketoamide derivatives from sulfoxonium ylides. Org. Lett..

[41] Nguyen T.B., Tran M.Q., Ermolenko L., Al-Mourabit A. (2014). Three-component reaction between alkynes, elemental sulfur, and aliphatic amines: A general, straightforward, and atom economical approach to thioamides. Org. Lett..

[42] Li W., Wu X., Zhao Z., Qin A., Hu R., Tang B.Z. (2015). Catalyst-free, atom-economic, multicomponent polymerizations of aromatic diynes, elemental sulfur, and aliphatic diamines toward luminescent polythioamides. Biomacromolecules.

[43] Xu K., Li Z., Cheng F., Zuo Z., Wang T., Wang M., Liu L. (2018). Transition-metal-free cleavage of C–C triple bonds in aromatic alkynes with S_8_ and amides leading to aryl thioamides. Org. Lett..

[44] Zhang P., Chen W., Liu M., Wu H. (2018). Base-controlled three component reactions of amines, elemental sulfur, and styrenes: Synthesis of thioamides under metal-free conditions. J. Org. Chem..

[45] Gan L., Gao Y., Wei L., Wan J.-P. (2019). Synthesis of α-keto thioamides by metal-free C=C bond cleavage in enaminones using elemental sulfur. J. Org. Chem..

[46] Xu H.H., Zhang X.G., Zhang X.H. (2020). Thioamidation of arylpropyne derivatives with sulfur and formamides for the synthesis of aryl propanethioamides. Asian J. Org. Chem..

[47] Peng L., Ma L., Ran Y., Chen Y., Zeng Z. (2021). Metal-free three-component synthesis of thioamides from β-nitrostyrenes, amines and elemental sulfur. Tetrahedron Lett..

[48] Jaiswal A., Sharma A.K., Singh K.N. (2021). Copper-catalyzed decarboxylative synthesis of α-ketothioamides using α,α-unsaturated arylcarboxylic acids, alicyclic secondary amines and elemental sulfur. Asian J. Org. Chem..

[49] Vankar J.K., Gupta A., Jadav J.P., Nanjegowda S.H., Gururaja G.N. (2021). The thioamidation of *gem*-dibromoalkenes in an aqueous medium. Org. Biomol. Chem..

[50] Nguyen T.T.T., Ngo Q.A., Koleski M., Nguyen T.B. (2021). The catalytic role of elemental sulfur in the DMSO-promoted oxidative coupling of methylhetarenes with amines: Synthesis of thioamides and bis-aza-heterocycles. Org. Chem. Front..

[51] Milen M., Ábrányi-Balogh P., Dancsó A., Keglevich G. (2012). Microwave-assisted synthesis of thioamides with elemental sulfur. J. Sulfur. Chem..

[52] Nguyen T.B., Ermolenko L., Al-Mourabit A. (2012). Efficient and selective multicomponent oxidative coupling of two different aliphatic primary amines into thioamides by elemental sulfur. Org. Lett..

[53] Kurpil B., Kumru B., Heil T., Antonietti M., Savateev A. (2018). Carbon nitride creates thioamides in high yields by the photocatalytic Kindler reaction. Green Chem..

[54] Joshi A., Kumar R., Semwal R., Rawat D., Adimurthy S. (2019). Ionic liquid catalysed aerobic oxidative amidation and thioamidation of benzylic amines under neat conditions. Green Chem..

[55] Zhou J., Wang S., Lu Y., Li L., Duan W., Wang Q., Wang H., Wei W. (2021). Solvent-driven C (sp^3^)–H thiocarbonylation of benzylamine derivatives under catalyst-free conditions. Green Chem..

[56] Li H.-Z., Xue W.-J., Wu A.-X. (2014). Direct synthesis of α-ketothioamides from aryl methyl ketones and amines via I_2_-promoted sp^3^ C–H functionalization. Tetrahedron.

[57] Wang C., Han C., Yang J., Zhang Z., Zhao Y., Zhao J. (2022). Ynamide-mediated thioamide and primary thioamide syntheses. J. Org. Chem..

[58] Yang J., Wang C., Xu S., Zhao J. (2019). Ynamide-mediated thiopeptide synthesis. Angew. Chem. Int. Ed..

[59] Wei J., Li Y., Jiang X. (2016). Aqueous compatible protocol to both alkyl and aryl thioamide synthesis. Org. Lett..

[60] Cao X.-T., Qiao L., Zheng H., Yang H.-Y., Zhang P.-F. (2018). A efficient protocol for the synthesis of thioamides in [DBUH][OAc] at room temperature. RSC Adv..

[61] Sun Y., Jiang H., Wu W., Zeng W., Li J. (2014). Synthesis of thioamides via one-pot A3-coupling of alkynyl bromides, amines, and sodium sulfide. Org. Biomol. Chem..

[62] Morri A.K., Thummala Y., Adepu R., Sharma G.V., Ghosh S., Doddi V.R. (2019). Synthesis of substituted thioamides from *gem*-dibromoalkenes and sodiumsulfide. Eur. J. Org. Chem..

[63] Zhou Z., Yu J.-T., Zhou Y., Jiang Y., Cheng J. (2017). Aqueous MCRs of quaternary ammoniums, *N*-substituted formamides and sodium disulfide towards aryl thioamides. Org. Chem. Front..

[64] Bergman J., Pettersson B., Hasimbegovic V., Svensson P.H. (2011). Thionations using a P_4_S_10_-pyridine complex in solvents such as acetonitrile and dimethyl sulfone. J. Org. Chem..

[65] Wang X., Ji M., Lim S., Jang H.-Y. (2014). Thiol as a synthon for preparing thiocarbonyl: Aerobic oxidation of thiols for the synthesis of thioamides. J. Org. Chem..

[66] Chen S., Li Y., Chen J., Xu X., Su L., Tang Z., Au C.-T., Qiu R. (2016). Iodine-promoted synthesis of thioamides from 1,2-dibenzylsulfane and difurfuryl disulfide. Synlett.

[67] Chen J., Mei L., Liu J., Zhong C., Yuan B., Li Q. (2019). Microwave-assisted iodine-catalyzed oxidative coupling of dibenzyl (difurfuryl) disulfides with amines: A rapid and efficient protocol for thioamides. RSC Adv..

[68] Rezapour M., Abbasi M. (2023). Oxidative coupling of dibenzyl disulfide with amines catalyzed by quinoline-bromine complex: Access to thioamides. Mol. Divers..

[69] Bian Y., Qu X., Chen Y., Li J., Liu L. (2018). K_2_S_2_O_8_-promoted aryl thioamides synthesis from aryl aldehydes using thiourea as the sulfur source. Molecules.

[70] Zhang Y., Ye X., Liu S., Chen W., Majeed I., Liu T., Zhu Y., Zeng Z. (2021). NaOTs-promoted transition metal-free C-N bond cleavage to form C-X (X= N, O, S) bonds. Org. Biomol. Chem..

[71] Li G., Xing Y., Zhao H., Zhang J., Hong X., Szostak M. (2022). Chemoselective transamidation of thioamides by transition-metal-free N-C(S) transacylation. Angew. Chem. Int. Ed..

[72] Lu C., Li X., Chang S., Zhang Y., Xing D., Wang S., Lin Y., Jiang H., Huang L. (2022). Thioamide synthesis via copper-catalyzed C-H activation of 1,2,3-thiadiazoles enabled by slow release and capture of thioketenes. Org. Chem. Front..

[73] Hegedus L.L., McCabe R.W. (1984). Catalyst Poisoning.

[74] Wu K., Ling Y., Ding A., Jin L., Sun N., Hu B., Shen Z., Hu X. (2021). A chromatography-free and aqueous waste-free process for thioamide preparation with Lawesson’s reagent. Beilstein J. Org. Chem..

